# γδ T Cells and Inborn Errors of Immunity

**DOI:** 10.1002/eji.202451457

**Published:** 2025-06-24

**Authors:** Sagar, Stephan Ehl

**Affiliations:** ^1^ Department of Medicine II (Gastroenterology, Hepatology, Endocrinology, and Infectious Diseases) Faculty of Medicine Freiburg University Medical Center University of Freiburg Freiburg Germany; ^2^ Institute for Immunodeficiency Center for Chronic Immunodeficiency Faculty of Medicine Medical Center‐University of Freiburg University of Freiburg Freiburg Germany; ^3^ CIBSS ‐ Cente for Integrative Biological Signalling Studies University of Freiburg Freiburg Germany

**Keywords:** γδ T cells, inborn errors of immunity, primary immunodeficiency, human, mouse

## Abstract

Gamma delta (γδ) T cells are pivotal in diverse immune responses, encompassing defense against infections, cancer surveillance, and tissue repair. Inborn errors of immunity (IEI) are rare genetic conditions disrupting human immune system development and function, with some impacting γδ T cells. In this review, we focus on IEI leading to a relative increase or decrease of γδ T cells compared to αβ T cells. We discuss how these disorders provide unique insights, in particular concerning the importance of signaling pathways for human αβ versus γδ T cell development, function, and homeostasis. Wherever suitable, we also include data from respective mouse models of IEIs that corroborate patient observations but also illustrate relevant species differences. This comparative approach identifies gaps in knowledge and defines areas for future research. Overall, this review underscores the relevance of IEIs in elucidating the development and function of human γδ T cells with potential implications for diagnosing and treating patients with immune disorders.

## Graphical abstract

This review examines inborn errors of immunity (IEIs) affecting γδ T cells, focusing on conditions that lead to their altered balance with αβ T cells. It highlights key molecular players and species differences in human and mouse models, offering insights into γδ T cell development and function with potential diagnostic and therapeutic implications.

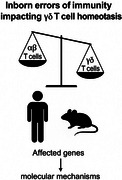

**Abbreviations**
AT: ataxia teleangiectasia
CMV: cytomegalovirus
IEI: inborn error of immunity
IEL: intraepithelial lymphocytes
NBS: Nijmegen breakage syndrome
RAG: recombinase activating gene
SCID: severe combined immunodeficiency
TCR: T cell receptor
αβ: alpha beta
γδ: gamma delta

## 1 Introduction

γδ T cells are a subset of T cells characterized by the expression of a unique T cell receptor (TCR) composed of γ and δ chains. These cells possess distinct antigen recognition properties and are involved in a wide range of immune responses, including host defense against infections, surveillance of cancer cells, and tissue repair [1]. Therefore, understanding the development and functions of γδ T cells is essential for advancing our knowledge of immune system function and improving strategies for treating infectious diseases and cancer. Inborn errors of immunity (IEI) are a group of rare disorders caused by genetic defects that impair the development and functions of the immune system and can affect its various components, including γδ T cells [2, 3]. Genetic mutations affecting the γδ T cell compartment in IEIs can provide valuable insights into their development and functions through the identification of key molecular players. They offer a unique opportunity to validate concepts generated in mouse models, which can be an important step when translating such concepts to therapeutic approaches in human disease.

γδ T cells differ significantly from αβ T cells in their antigen recognition, development, and function. They can recognize antigens directly without MHC presentation, allowing for a broader range of targets including nonpeptidic molecules and stress‐induced antigens. γδ T cells often reside in epithelial tissues, can respond rapidly without clonal expansion, and bridge innate and adaptive immunity with their ability to act quickly while also forming memory‐like responses [4]. Their unique TCR properties, including limited diversity in some subsets, and their capacity for single‐signal activation further distinguish them from conventional αβ T cells, highlighting their specialized roles in early immune defense and tissue homeostasis [5]. For this review, we decided to focus on human genetic conditions that lead to a relative increase or decrease of γδ T cells compared with αβ T cells. In this context, IEI can be informative concerning the differential importance of signaling pathways for human αβ versus γδ T cell development, function, and homeostasis. Clinical and immunological phenotypes in immunodeficient patients can be significantly modified by infectious diseases or immunomodulatory treatment. Relating human observations to mouse studies is therefore essential since this allows us to identify and control such nongenetic factors. However, a comparison of human and mouse studies may also uncover relevant species differences. We are convinced that a comparative approach can lead to more solid conclusions about human γδ T cell immunity and can identify gaps of knowledge that should be explored in the future.

**FIGURE 1:**
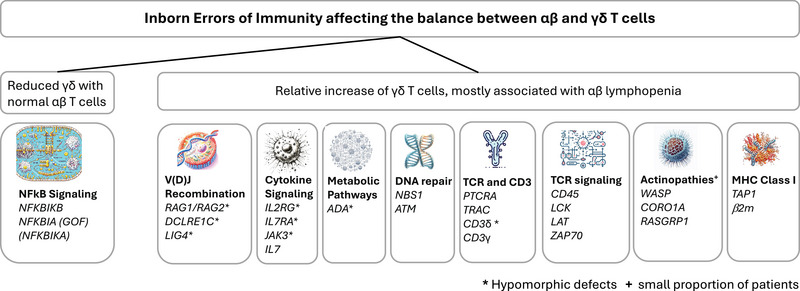
Summary of human genetic conditions associated with an altered balance between αβ and γδ T cells. * indicates genes in which hypomorphic, but not null mutations have been associated with γδ T cell predominance. + indicates genes in which mutations have been associated with γδ predominance only in a few patients.

With almost 500 molecularly defined IEI [2], a comprehensive review of altered γδ T cell immunity in IEI is a significant challenge. In many publications on IEI patients and corresponding mouse models, no particular attention has been paid to γδ T cells. Moreover, PubMed searches for γδ T cells are notoriously difficult. These difficulties necessarily render this review incomplete and we apologize to all authors whose work has not been considered. As a basis for this manuscript, we screened the literature using search terms “γδ” or “gamma delta”, “immunodeficiency not HIV”, and “inborn error of immunity” and screened these papers, their references, and references quoting these papers. If a certain disease group was identified (e.g., disorders of NF‐kB immunity), we also screened the literature on other diseases from this group. Finally, we included mouse models of the human condition wherever feasible (Figure 1).

### 1.1 IEI With Reduced or Absent γδ T Cells in the Presence of αβ T Cells

Several patients with loss‐of‐function mutations in nuclear factor kappa B inhibitor kinase subunit beta (NFKBIKB) or gain‐of‐function mutations in nuclear factor kappa B inhibitor alpha (NFKBIA) were reported to have absent γδ T cells in the presence of αβ T cells [6, 7, 8, 9]. Furthermore, low γδ T cells (2%) were found in a single patient with nuclear factor kappa B inhibitor kinase subunit alpha (NFKBIKA) deficiency. In these conditions, canonical NF‐kB signaling is impaired because Ubiquitin‐mediated degradation of IKB is defective. IKB remains bound to NF‐κB, inhibiting its function. This results in impaired αβ T cell activation leading to their impaired proliferation, and differentiation into effector and memory cells. However, while γδ T cells are absent, these patients do not have significant αβ T cell lymphopenia. In mice, NFKBIKB deficiency is embryonically lethal [11] and T cell‐specific deletion of NFKBIKB leads to impaired regulatory and memory T cell differentiation in the αβ T cell compartment [12]. However, γδ T cells were not investigated in this study. Reduced γδ T cells were not reported in patients with other defects of the NF‐kB pathway including defects of the CBM (CARD11, BCL10, MALT1) complex [13], A20 (TNFAIP3) haploinsufficiency [14], NEMO (IKBKG) deficiency [15], and NFKB1 or NFKB2 haploinsufficiency [16, 17]. It is noteworthy, however, that patients with NEMO deficiency carry hypomorphic variants, compatible with mouse data showing that complete NEMO deficiency is embryonically lethal [18]. This may explain why human NFKBIKB deficiency, but not (hypomorphic) NEMO mutations lead to absent γδ T cells.

Taken together, these observations point to a specific role for NFkB signaling, and in particular a stable and functional IKK complex for the development and/or maintenance of γδ T cells, which should be further explored in humans and mice.

## 2 IEI With Normal or Increased γδ T Cells in the Presence of Reduced αβ T Cells

### 2.1 “Atypical” Severe Combined Immunodeficiency

Severe combined immune deficiency (SCID) is a life‐threatening primary immunodeficiency with a combined absence of T cell and B cell function, in most cases due to mutations in genes that are essential for T cell development [19]. They mainly include genes involved in VDJ recombination such as RAG1, RAG2, DCLRE1C, or LIG4, and genes involved in cytokine signaling including IL2RG, IL7R, or JAK3. SCID can also result from severe defects in thymic development (e.g., 22q11.2 deletion or DiGeorge syndrome, CHD7 deficiency or CHARGE syndrome, TBX1 or FOXN1 deficiency) or from ADA1 deficiency, a systemic disorder of purine metabolism primarily affecting lymphocyte development, viability, and function. Null mutations in these genes cause “classical” SCID and are associated with absent αβ and γδ T cells. In contrast, hypomorphic mutations in these genes leading to residual function of the affected protein allow variable degrees of T cell development. These conditions have been termed “atypical” SCID [20, 21] based on (1) functionally relevant mutation(s) in a SCID‐associated gene and (2) at least two of the following four criteria: low T‐cell numbers, oligoclonal T cells, low T‐cell receptor excision circles, and less than 20% naive CD4 T cells.

Increased proportions of γδ relative to αβ T cells were first specifically reported in two patients with hypomorphic RAG mutations [22, 23]. Both patients suffered from persistent CMV infection, which is known to stimulate γδ T cell expansion. However, γδ T cell predominance in RAG‐deficient patients was also reported in the absence of CMV infection [24]. Furthermore, mice with engineered patient‐derived hypomorphic mutations in RAG1 had an increased proportion of thymic γδ T cells [25]. Unfortunately, the proportions of γδ lymphocytes in the periphery were not reported. Thus, reduced RAG activity appears to affect γδ T cells less than αβ T cells both in humans and in mice. This may be linked to differences in RAG expression in the two cell types. While cells differentiating into γδ thymocytes only show a single period of RAG expression to rearrange *TRG* and *TRD* αβ T cells transiently downregulate *Rag* expression after successful rearrangement of *TRB* and then show a second wave of RAG expression at the CD4^+^CD8^+^ double‐positive (DP) stage to rearrange the *TRA* locus [26]. The lower number of V(D)J segments at the *TRG/TRD* locus may also require less RAG activity.

Importantly, elevated proportions of γδ T lymphocytes are not a specific feature of RAG1/2 associated “atypical” SCID but have also frequently been observed in patients with other hypomorphic mutations in SCID genes. In a literature review of 76 patients with “atypical” SCID and available information on γδ T cells, 43 displayed proportions of γδ T lymphocytes above 15%, irrespective of whether patients had recombination defects, cytokine receptor defects, or ADA1 deficiency [27]. These observations could indicate that γδ T cells are generally less vulnerable to (partial) defects affecting intrinsic T cell development than αβ T cells. This interpretation is further supported by the recently described human IL‐7 deficiency, which is associated with profound αβ T cell lymphopenia, while γδ T cells are little affected [28]. In one atypical IL2RG deficient patient, γδ T cell expansion was associated with a somatic second‐site mutation restricted to γδ T cells, pointing out an additional factor that can contribute to γδ T cell predominance in these patients [29].

Interestingly, the proportion of γδ T cells is rather reduced in patients with severe but incomplete thymic hypoplasia [30]. This may suggest an advantage for αβ T cell development under conditions of a limited thymic niche, in contrast to conditions of T cell‐intrinsic developmental defects.

Future studies on γδ T cells in human “atypical” SCID should benefit from the widespread implementation of newborn screening, allowing the identification of patients without prior infection, which can be an important confounder. Indeed, in the “atypical” γδ T cell SCID study, CMV infection was more prevalent in patients with elevated than in those with normal proportions of γδ T cells [27], consistent with the observation that CMV is an important trigger of γδ T cell expansion in particular under conditions of αβ T cell lymphopenia [31].

### 2.2 Combined Immunodeficiencies With TCR Signaling Defects

Defects of the αβ TCR itself, the associated CD3 subunits, or signaling molecules proximal to the TCR can lead to profound T cell lymphopenia. The impact of such defects on γδ T cell versus αβ T cell immunity informs about potential differences in signaling requirements for the development and maintenance of these two T cell populations. The phenotype of patients with isolated absence of αβ T cell development may also inform about the compensatory potential of γδ T cell immunity during antimicrobial immune responses.

In two genetic conditions affecting the TCR components, αβ T cell development was found abolished or reduced without affecting γδ T cell development. Ten patients have been described with complete pre‐TCRα (PTCRA) deficiency, impairing αβ, but not γδ T cell development [32]. However, these patients develop αβ T cells via non‐canonical pathways, resulting in a lower thymic output, but eventually normal counts of memory αβ T cells. It was found that TCRα rearrangements were highly biased toward TCRδ1 rearrangements. However, these αβ T cells did not derive from γδ thymocytes. The exact mechanism through which αβ T cell differentiation is rescued and the potential role of the TCRδ chain in this process remain unclear [32]. γδ T cell numbers are elevated in patients with PTCRA deficiency with a normal balance between Vδ1 and Vδ2 subsets. The phenotype of these patients was surprisingly mild with no significant infections, lymphoproliferation, or autoimmunity before adolescence. Mice lacking the *Ptcra* gene exhibit a similar phenotype, where γδ T cell development remains unaffected [33]. Furthermore, so far five patients have been reported with mutations in the TCR alpha subunit constant gene (TRAC deficiency) [34, 35]. In these individuals, all T cells expressing normal levels of CD3 were γδ T cells. In addition, however, patients also had an unusual population of CD3^lo^ cells expressing TCRαβ at a very low level. The patients showed features of a combined immunodeficiency with severe viral and bacterial infections in childhood, associated with immune dysregulation including lymphoproliferation, eczema, eosinophilia, elevated IgE, and autoimmunity. However, they were able to generate specific antibodies (including autoantibodies) and did not show a full SCID phenotype [34, 35].

The observations in these two IEI are consistent with observations in the respective knock‐out mice and show that the pre‐TCR alpha is not required for human γδ T cell development [33, 36]. Due to the residual atypical αβ T cells in both conditions, they do not represent a clean model situation in which only γδ T cells can provide T cell immunity. Therefore, the observation of a less severe “profound CID” rather than a full “SCID” phenotype (i.e., lethality in the first 1–2 years of life in the absence of therapeutic T cell reconstitution) in TRAC deficient patients cannot easily be interpreted as evidence for a partial compensatory role of γδ T cells in infection control.

Deficiencies in the components of the CD3 complex have also been associated with specific consequences for αβ versus γδ T cell immunity. Complete human CD3δ deficiency results in the absence of T cells [37]. Mice lacking the CD3δ subunit exhibit a specific intrathymic developmental arrest of αβ T cells, while γδ T cell development is not affected, and normal levels of γδ T cells in the peripheral organs are observed [38]. In contrast, human partial CD3δ deficiency is associated with strongly reduced αβ but normal numbers of γδ T cells with a strong enrichment of CD4+ γδ T cells [39]. These γδ T cells, however, show low surface TCR expression resulting in impaired function. The combination of developmental and functional T cell defects leads to a severe SCID phenotype in affected patients. Complete human CD3γ deficiency impairs the surface expression of CD3 and TCR molecules, reducing TCR signaling strength [40]. T cell numbers including γδ T cells can be normal in these patients, but moderate T cell lymphopenia develops with age and γδ T cells show an abnormal γδTCR/δεδεζζ stoichiometry [41]. In contrast, in mice the CD3δ chain cannot replace the CD3γ chain and CD3γ‐deficient mice exhibit a severe block in γδ T cell development [42]. Overall, these findings indicate important species differences in the requirement of CD3 signaling for T‐cell development.

### 2.3 Combined Immunodeficiencies with Defects of Proximal TCR Signaling Components

Several human deficiencies of proximal signaling components of the TCR have been associated with γδ T cell predominance. CD45 is a transmembrane protein tyrosine phosphatase that controls the threshold of sensitivity to external stimuli by regulating Src kinases required for T‐cell signal transduction. CD45 deficiency leads to severe T cell lymphopenia with preserved γδ T cells [43, 44]. However, these γδ T cells are poorly functional since γδ TCR triggering by nonclassical MHC class Ib antigens requires steric segregation of CD45 from engaged TCRs at synaptic close‐contact zones [45]. CD45 deficient patients develop a full SCID phenotype. All four reported patients with LCK deficiency presented with severe T cell lymphopenia with an increased proportion of γδ T cells [46, 47, 48]. Mice lacking LCK exhibit drastic thymic atrophy and also possess very few peripheral T cells. However, unlike in humans, γδ T cells are significantly reduced in the spleen and lymph nodes of these mice [49, 50]. The role of LCK in nonlymphoid organs has also been explored revealing that while mouse epidermal γδ T cells, known as dendritic epidermal T cells (DETCs) are also reduced in LCK deficient mice while the intraepithelial γδ T cell compartment in the intestine remains unaffected [51, 52]. Complete LAT deficiency was also associated with γδ T cell predominance in two patients [53]. LAT‐deficient mice display a block in αβ and γδ T cell development, as well as the absence of both lineages in the periphery [54]. However, mice homozygous for a mutation of the three C‐terminal LAT tyrosine residues had partial γδ T cell development in the absence of αβ T cells. They accumulated γδ T cells in lymphoid tissue that produced excessive Th2 cytokines leading to elevated IgE [55]. Finally, patients with ZAP70 deficiency have nonfunctional CD4+ T cells at normal levels and develop severe or progressive CD8 lymphopenia [56]. Interestingly, among the residual CD8 T cells, there is a large predominance of CD8+ γδ T cells [57]. In mice, ZAP70 in the γδ T cell compartment seems to be required only for the differentiation of IL‐17‐producing γδ T cells, the presence of which is still debated in humans, making a direct comparison difficult [58].

Overall, these findings could be interpreted to indicate that defects in proximal TCR signaling affect γδ T cell development less than αβ T cell development. This is in contrast to data from mice, where weaker signals have been shown to promote αβ T cell fate [59, 60]. However, a note of caution is justified with the human data, since it cannot be excluded that the γδ predominance is not developmental, but a result of peripheral events including homeostatic proliferation or infections that lead to peripheral γδ T cell expansion.

### 2.4 Combined Immunodeficiencies Due to DNA Repair Defects

Two DNA repair defects have been associated with increased proportions of γδ T cells in the context of T cell lymphopenia, that is, Nijmegen breakage syndrome (NBS) and ataxia telangiectasia (AT) [61, 62, 63]. NBS is caused by mutations in the NBS1 gene encoding a protein called nibrin, which plays a crucial role in the repair of DNA double‐strand breaks (DSBs) during the process of nonhomologous end joining [64]. Nibrin interacts with other proteins involved in DNA repair, such as MRE11 and RAD50, forming the MRN complex [65]. This complex is essential for sensing and signaling DNA damage, as well as facilitating repair processes. NBS1 predominantly co‐localizes in foci with the TCRA locus in developing thymocytes [66]. This indicates that the TCRG/TCRD rearrangement may be less sensitive to a recombination defect than TCRB/TCRA rearrangements, which may explain the γδ T cell predominance in NBS patients. AT is caused by mutations in the ATM gene, which plays a crucial role in sensing and repairing DNA damage, particularly DSBs. Aberrant TCRδ rearrangements have been identified as a cause for αβ T‐cell lymphopenia [67].

Mice with mutations in the NBS1 or ATM genes recapitulate many features of NBS and AT patients, including immunodeficiency and a heightened risk of lymphoid malignancies [68, 69]. Both models show reduced thymic cellularity and a lower number of αβ T cells in the periphery, but data on γδ T cells have not been reported to our knowledge, leaving room for further studies.

### 2.5 Combined Immunodeficiencies Due to Actin‐Related IEI

An increased proportion of γδ T cells has also been observed in several but not all patients with immunodeficiencies characterized by aberrant cytoskeletal dynamics, including Coronin‐1A deficiency [70], RASGRP1 deficiency [71], and Wiskott–Aldrich syndrome [72, 73]. Coronin‐1A regulates T cell trafficking and migration within lymphoid organs and tissues, as well as T cell receptor signaling and immune synapse formation during T cell activation [74]. We did not find data on the γδ T cell compartment of Coronin‐1A deficient mice. RASGRP1 functions as a guanine nucleotide exchange factor for Ras proteins and activates them by catalyzing the exchange of guanosine diphosphate for guanosine triphosphate. RASGRP1 is involved in TCR‐Ras‐Erk signaling pathways that regulate T cell activation, differentiation, and cytokine production [75]. Mouse studies showed that RASGRP1 is required for the development of αβ T cells, but plays a minimal role in γδ T cell development [76], leading to an increased number of γδ T cells in the peripheral lymphoid organs in mice. Mutations in the WAS gene that result in the loss or dysfunction of the Wiskott–Aldrich syndrome protein (WASp) impair TCR signaling and T cell activation leading to impaired proliferation, cytokine production, and cytotoxicity. The differential roles of WASP in the αβ and γδ T cell compartments have not been explored in WASP‐deficient mice [77, 78]. However, T‐cell‐specific replacement of the actin remodeling protein Cofilin by a nonfunctional version leads to a complete developmental defect of αβ, but not of γδ T cells [79]. Potential explanations offered to explain this phenotype include (1) a diminished migratory capacity of early thymocytes, which may be more essential for αβ T cell maturation, (2) a reduced clustering and distribution of surface receptors, for example, during synapse formation, which differs between αβ and γδ T cells, and (3) the need for cofilin‐driven processes for cell surface expression of TCRβ, but less for γδ TCR upregulation. Such considerations may also apply to other actin‐related proteins, but their specific role in αβ versus γδ T cell homeostasis remains to be defined.

### 2.6 Combined Immunodeficiencies Due to MHC Class I Deficiency

Human MHC class I deficiency is caused by loss‐of‐function mutations in TAP1 or β2 microglobulin [80]. Patients show low CD8+ but normal CD4+ αβ T cells. Interestingly, a predominance of CD8+ γδ T cells has been observed in these patients [81]. Investigations on γδ T cells in mouse models of TAP1 or β2M deficiency have focused on the analysis of intraepithelial lymphocytes (IEL) [82]. While CD8αα+ and CD8αβ+ αβ ‐IEL subsets were drastically reduced, there was a compensatory two‐ to threefold increase in the number of γδ IEL, consisting mostly of CD8 αα+ subset [83]. Overall, the normal development of γδ T cells in MHC I and MHC II deficient patients and mice is in line with the concept that they are generally not MHC‐restricted.

## 3 Conclusion and Future Perspectives

In conclusion, the comparative study of IEI and corresponding mouse models offers a unique perspective for unraveling the complex biology of γδ T cells in humans. The genetic conditions summarized in this review focus on signaling pathways that illustrate differences between αβ and γδ T cell immunity. They illustrate overlapping pathways between humans and mice but also point out differences. While some of these differences can be explained by nongenetic factors or species differences, others remain unresolved and identify important areas for research. Looking ahead, further research into IEI and their impact on γδ T cells has much more to offer. In‐depth mechanistic analysis of γδ T cells obtained from IEI patients holds significant promise for advancing our molecular understanding of γδ T cell immunity in humans. With the advances in human genetics and the advent of newborn screening, the identification of patients with IEI affecting γδ T cells has become more efficient. The increasing recognition of human immune diseases caused by somatic mutations offers additional opportunities [84]. Continued investigation of γδ T cells in such patients will yield further insights into the signaling pathways and molecular mechanisms governing γδ T cell development and function, potentially uncovering new targets for therapeutic intervention.

## Author Contributions

Stephan Ehl reviewed the clinical data, and Sagar reviewed the corresponding data from murine models. Both authors discussed and interpreted the combined human and murine evidence.

## Conflicts of Interest

The authors declare no conflicts of interest.

## 1 Peer Review

The peer review history for this article is available at https://publons.com/publon/10.1002/eji.202451457.

## Data Availability

All data generated or analyzed during this study are included in this article.
